# Monoclonal antibody-based colloid gold immunochromatographic strip for the rapid detection of *Tomato zonate spot tospovirus*

**DOI:** 10.1186/s12985-018-0919-5

**Published:** 2018-01-18

**Authors:** Yanbing Niu, Defu Wang, Liyan Cui, Baoxia Wang, Xiaojing Pang, Peixia Yu

**Affiliations:** College of Life Sciences, Shanxi Agricultural University, Taigu, 030801 China

**Keywords:** *Tomato zonate spot virus* (TZSV), Monoclonal antibodies, Colloid gold immunochromatographic (GICA) strip, Rapid detection

## Abstract

**Background:**

*Tomato zonate spot virus* (TZSV), a new species of genus *Tospovirus*, caused significant losses in yield and problems in quality of many important vegetables and ornamentals in Southwest China and posed a serious threat to important economic crops for the local farmers. A convenient and reliable method was urgently needed for rapid detection and surveillance of TZSV.

**Methods:**

The nucleocapsid protein (N) of TZSV was expressed in *Escherichia coli* and purified, and was used as the antigen to immunize BALB/c mice. Three monoclonal antibodies (mAbs) 3A2, 5D2 and 5F7 against TZSV were obtained through the hybridoma technique. The mAb 3A2 was conjugated with colloid gold as detecting reagent; mAb 5D2 was coated on a porous nitrocellulose membrane as the detection line and protein A was coated as the control line respectively. The colloid gold immunochromatographic (GICA) strip was assembled.

**Results:**

The analysis of Dot-ELISA and Western blot showed that the obtained three independent lines of mAbs 3A2, 5D2 and 5F7 specifically recognized TZSV N. Based on the assembly of GICA strip, the detection of TZSV was achieved by loading the infected sap onto the test strip for visual inspection. The analysis could be completed within 5–10 min. No cross-reaction occurred between TZSV and other tested viruses. The visual detection limit of the test strip for TZSV was 800 fold dilutions of TZSV-infected leaf samples.

**Conclusion:**

The mAbs were specific and the colloidal GICA strip developed in this study was convenient, fast and reliable for the detection of TZSV. The method could be applied for the rapid diagnosis and surveillance of TZSV in the field.

## Background

Tospoviruses are the most devastating plant viruses and caused significant losses in many important economic crops throughout the world [1–3]. The diseases caused by tospoviruses spreaded rapidly in Yunnan province, Southwest China [4–12]. Many species of tospoviruses were reported in China. *Tomato zonate spot virus* (TZSV) was one of main local tospoviruses in Yunnan province and was characterized by Dong et al. 2008 [7]*.* The virus has round, enveloped particle and is transmitted by thrip in a propagative manner [7, 13]. The infection of TZSV is often associated with severe diseases including concentric zoned ringspots on fruits and necrotic lesions on leaves [7]. The host range of TZSV was extensively characterized. It infected more than 20 plants species belonging to 7 families, including economic crops and weeds [14, 15]. It posed a serious threat to the tomato, tobacco and orgmental productions for local farmers in Southwest China [16, 17]. A rapid, convenient and practical method was urgently needed to detect and prevent further spread of TZSV.

TZSV belongs to genus *Tospovirus* in the family of *Bunyaviridae* [18, 19]. Similar to other members in genus *Tospovirus*, TZSV has tripartite-segmented single-stranded RNA (ss RNA) genome, named as large (L), medium (M) and small (S) RNAs [7, 20]. The L RNA is in negative sense and encodes a RNA-dependent RNA polymerase (RdRp) (2885 aa, 332.7 kDa) for replication and transcription [21, 22]. The M RNA encodes a non-structural (NSm) protein (309 aa, 34.4 kDa) from the viral sense and the envelope glycoproteins Gn and Gc (1122 aa, 127.4 kDa) from the viral complementary sense [23–25]. The S RNA encodes a suppressor of plant gene silencing (NSs) (459 aa, 51.9 kDa) from the viral sense and a nucleocapsid (N) (278 aa, 30.6 kDa) protein from the viral-complementary sense [26–28].

Tospoviruses can be identified by enzyme-linked immunosorbent assay (ELISA) [29, 30], electron microscopy [31, 32], and reverse transcription polymerase chain reaction amplification (RT-PCR) [33–37]. However, all these detection methods need special equipment and do not meet the requirement of rapid or on-site detection. The colloidal gold immunochromatographic assay (GICA) is a simple and rapid detection method. The analysis can be completed in 5–10 min without sophistic equipment. It has been used for diagnosis of animal viral diseases including porcine reproductive and respiratory syndrome virus [38], porcine circovirus-2 [39], avian influenza virus and some plant viruses. GICA strip for the rapid detection of *Soybean mosaic virus *(SMV) [40] have been successfully developed.

In this study, we immunized mice using purified recombinant nucleocapsid (N) of TZSV and obtained three mAbs. Based on mAbs, we developed a GICA strip for the rapid detection of TZSV. The analysis in test strip could be completed within 5–10 min. The detection was specific for TZSV and it had no cross reaction with other tested tospoviruses or unrelated plant viruses, and the sensitivity of strip also met the detection requirements. It had a potential application prospect in the prevention of TZSV dissemination.

## Methods

### Expression and purification of TZSV-N

The fragment of TZSV *N* gene was amplified from the cDNA of TZSV-infected tomato plants (Genbank accession number EF552434.1) using the primers XT666 (5′-CGGGATCCATGTCTAACGTCCGGAGTTT-3′, the restriction site *Bam*H I is underlined) and XT667 (5′- CCGCTCGAG*TTA*AAAAGACAGATCATTGCTG-3′, the restriction site *Xho* I is underlined). The recovery PCR products were digested with *Bam*H I and *Xho* I and inserted into pET28a digested with the same restriction enzymes to generate the construct pET28a-TZSV-N. The positive clone was confirmed by sequence and then transferred into *Escherichia coli* expression strain *Rossetta* (DE3).

The *Rossetta* cells containing pET28a-TZSV-N were cultured in 5 mL of LB medium containing 50 μg/mL kanamycin and 17 μg/mL chloromycetin at 37 °C for 8 h. One mL of the culture was transferred into 1 L of LB medium containing 50 μg/mL kanamycin and 17 μg/mL chloromycetin at 37 °C to reach the optical density at 600 nm (OD600) of 0.6, 1 mL of 0.1 mM IPTG was added into the culture, which was then incubated at 200 r/min at 20 °C for about 10 h. The culture was centrifuged at 8, 000 r/min for 5 min and the pellet was resuspended with 40 mL lysis buffer (10 mM imidazole, 50 mM NaH_2_PO_4_ and 300 mM NaCl, pH 8.0), subsequently 40 μL 100 mg/mL of lysozyme was added and placed in ice for 30 min. After ultrasonic fragmentation, the culture was centrifuged at 10, 000 r/min for 70 min at 4 °C. The supernatant was mixed with 1 mL Ni-NTA resin, followed by shaking incubation for 2 h at 4 °C. The mixed solution was centrifuged at 3, 100 r/min for 5 min and the precipitation retain was washed with 10 mL of 20 mM wash buffer (20 mM imidazole, 50 mM NaH_2_PO_4_ and 300 mM NaCl, pH 8.0) and 10 mL of 50 mM wash buffer (50 mM imidazole, 50 mM NaH_2_PO_4_ and 300 mM NaCl, pH 8.0) for 3 times separately. The precipitation was transferred into the purification column, then 10 mL of 50 mM wash buffer was added to wash it. The target protein was eluted and collected from the filter membrane of the column with Elution buffer (250 mM imidazole, 50 mM NaH_2_PO_4_ and 300 mM NaCl, pH 8.0), and its concentration was measured using an ultraviolet spectrophotometer (NanoDrop2000). The purified recombinant protein was dialysed into 1 × PBS to remove the imidazole.

### Sodium dodecyl sulfate-polyacrylamide gel (SDS-PAGE) and western blot

Ten μL target protein was resuspended with 5 μL of 3 × SDS loading buffer, boiled for 10 min, and the boiled protein sample was separated by electrophoresis in 12.5% SDS-PAGE and stained with coomassie brilliant blue. For western blot, the protein was transferred onto an Nitrocellular (NC) membrane (Whatman, Dassel, Germany). The NC membrane was blocked with 20 mL of 5% milk in PBST (PBS containing 0.05% (*v*/v) Tween-20, pH 7.4) at 28 °C for 45 min. 10 mL of antiserum diluted with 5% milk in PBST (1:5000) was added to cover the NC membrane, or the NC membrane was incubated at 28 °C for 1.5 h with anti-TZSV-N mAbs. After washing for three times, 10 mL of alkaline phosphatase conjugated secondary antibody (Sigma, Shanghai, China) (1:30,000 in 5% milk in PBST) was added to cover the NC membrane for 1.5 h at 28 °C. The NC membrane was washed three times, and then 45 μL of NBT and 35 μL BCIP (Sangon, Shanghai, China) was added to cover the NC membrane for color development.

### Immunisation and screening of antisera

The purified recombinant TZSV-N was mixed with an equal volume of Freund’s complete adjuvant by repeated stirring to prepare the water-in-oil emulsion. The emulsion (containing 200 μg antigen protein per mouse) was injected into the peritoneal cavity of the 6-week-old BALB/c female mice. Twenty one days later, the purified recombinant TZSV-N was mixed with an equal volume of incomplete Freund’s adjuvant and then injected into the mice every 14 days, this operation was repeated for four times. Seven days after the last immunization, antiserum samples were obtained from the tail vein of each mouse. The antisera was tested for the titers and specificity to virus by indirect ELISA. The titers of the antisera from the five mice were determined by measuring the binding of serial dilutions of the antisera to the corresponding antigen (TZSV) using indirect ELISA.

### Indirect ELISA

Flat-bottomed polystyrene microtitre plates (Corning, New York, USA) were coated with crude extracts of TZSV-infected leaves and incubated at 4 °C for 8 h. The coated plates were washed once with PBST and blocked with 200 μL of 5% milk in PBST at 37 °C for 1 h. After one wash, 50 μL of antiserum diluted with 5% milk in PBST (1:100–1:320,000) was added to each well of the plates, then the plates were incubated at 37 °C for 1.5 h with cognate antibodies. After three times of washing, 50 μL of alkaline phosphatase conjugated secondary antibody (Sigma, Shanghai, China) (1:30,000 in 5% milk in PBST) was added to each well of the plates, and incubated for 1.5 h at 37 °C. The plates were washed three times, and then 50 μL of the 1 mg/mL pNPP-Na (Sangon, Shanghai, China) was added to each well of the plates for color development. The color development was stopped by 0.1 M EDTA (50 μL per well, pH 7.5). The absorbance was measured in single wave length mode at 405 nm within 30 min by a microplate reader (Biorad, Shanghai, China).

### Cell fusion and hybridoma selection

The mouse that showed the highest titer of the antiserum was selected to prepare the lymphocytes from the spleen. SP2/0 myeloma cells were cultured in high-glucose DMEM added with 20% fetal bovine serum (s-DMEM). The spleen lymphocytes were fused with SP2/0 myeloma cells at a ratio of 1:5 under the agent of the PEG 1500. The fused cells were distributed in the 96-well culture plates at an approximate density of 4 × 10^4^ cells/μL of HAT per well. Cells were grown in HAT medium for 14 days, then HAT was substituted by HT medium. One week to ten days later, the supernatants from these clones were screened and TZSV was recognized by indirect ELISA. The selected positive hybridoma cell lines were subsequently subcloned by limiting dilution method.

### Preparation, purification and isotyping of immunoglobulin

The hybridoma cell lines were injected into the BALB/c mice by intraperitoneal (Ip) injection of 2 × 10^7^ hybridoma cells. The ascites fluid was collected after 7 to 10 days of the injection. The immunoglobulin subclass and light chain isotype of the antibodies were determined using a Mouse Monoclonal Antibody Isotyping kit (Sigma, Shanghai, China). The mAb was purified with a protein A spin kit (Thermo, Beijing, China) according to the manufacturer’s instructions.

### Colloidal gold preparation

A 100 mL solution of 0.01% gold chloride was boiled to reflux station, then 1.1 mL of 1% tri-sodium citrate solution was added rapidly with constant stirring. The solution was boiled for additional 5 min until the color of the mixture changed to a brilliant wine red. After the solution was cooled at room temperature, and the average particle diameter was checked with a transmission electron microscope (TEM). The gold colloidal solution was added with 0.05% sodium azide and then stored at 4 °C.

### Conjugation of anti-TZSV mAb with colloidal gold

The colloidal gold (125 μL) was mixed with a series of gradiently diluted purified mAb 3A2, each gradient was added with 125 μL 10% NaCl. As the proportion of the antibodies increased, the color of the fixed solution gold would change from light to deep purple. The gradient whose color began to be stable had the optimal concentration. To conjugate anti-TZSV MAb with colloidal gold, the selected diluted mAb 3A2 were rapidly added into 20 mL of the colloidal gold solution (pH 8.2) with rapid stirring, then the solution was incubated at room temperature for about 1 h. The mixture was stabilized with 10% BSA solution (the final concentration of BSA was 1%) and stirred for 5 min at least, then was incubated for 1 h. The solution was centrifuged (10,000 r/min) at 4 °C for 25 min, the colorless supernatant was removed, and the loose sediment of gold-labeled mAb was resuspended with 20 mL of 2% BSA solution (containing 0.01 M sodium borate), the rest unbound mAb was removed through two or more round of centrifugations (10,000 r/min at 4 °C for 25 min). The obtained loose sediment of gold-labeled mAb was then resuspended with 4 mL of TB solution (containing 3% BSA, 3% sucrose, 0.01 M sodium borate and 0.05% sodium azide), and the final product was stored at 4 °C.

### Assembly of the GCIA strip

The GCIA strip was composed of a mAb-gold conjugated pad, a sample pad, an absorbent pad and an NC membrane. The pads and the NC membrane were all pasted onto an adhesive plastic backing. The NC membrane was pasted at the center of the backing plate. The mAb-gold conjugate pad was pasted by overlapping 1 mm on the bottom of the NC membrane, the sample pad was pasted by overlapping 2 mm on the bottom of the mAb-gold conjugate pad and the absorbent pad was pasted by overlapping1 mm on the upper position of the NC membrane. The whole assembled one-step strip was cut lengthways into 4.08-mm-wide strips using the guillotine cutter.

### Sensitivity and specificity of the GCIA

A series of dilutions from TZSV-infected *N. benthamiana* leaf extracts were tested using the GCIA strip. The extract from TZSV-uninfected leaves was used as the negative control. CMV, TMV, SMV, TSWV and INSV were used to test the specificity of the GCIA.

## Results

### Expression and purification of the recombinant TZSV-N protein

**Fig. 1:**
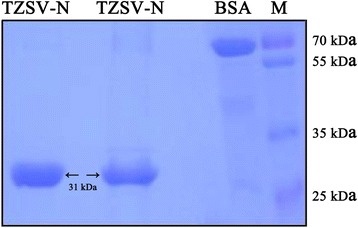
Expression and purification of the nucleocapsid protein of *Tomato zonate spot virus*. The recombinant TZSV N protein was expressed in Rosetta (DE3) and purified by Ni-NTA resin. The purified protein was separated on a 10% SDS-PAGE gel. The position of a 31-kDa recombinant N protein in the gel was indicated by an arrow. BSA and protein size marker were used as molecular weight reference

To obtain a sufficient amount of TZSV antigen for immunization, the full-length TZSV *N* was amplified from the total RNA extracted from TZSV-infected tomato plant by RT-PCR, the generated fragment was then cloned into the prokaryotic expression vector pET28a to produce the recombinant vector pET28-TZSV-N. The positive clone was sequenced and transformed into *E. coli* strain *Rossetta* (*DE3*). After isopropyl beta-D-thiogalactoside (IPTG) induction, the 6 x His-tagged recombinant protein TZSV N was purified by Ni-NTA resin. A SDS-PAGE analysis showed that the predicted 35-kDa protein was expressed and collected in the elution buffer containing 250 mM imidazole (Fig. 1). Finally, more than 2.5 mg (1 mg/mL in a 1 mL volume) of the recombinant TZSV N protein was obtained for immunization.

### Preparation of monoclonal antibodies against TZSV N

To produce specific mAbs against TZSV N, five 6-week-old BALB/c female mice were immunized with the purified TZSV N protein. After the fifth immunization, antiserum of one of the five mice showed the highest titer (1: 40,000) against crude extract from TZSV-infected *Nicotiana benthamiana* leaves. The spleen cells from this mouse were obtained for fusing with SP2/0 myeloma cells and then plated on six 96-well cell culture plates. After several rounds of subcloning and screening, three hybridoma lines 3A2, 5F7 and 5D2 were obtained.

**Fig. 2:**
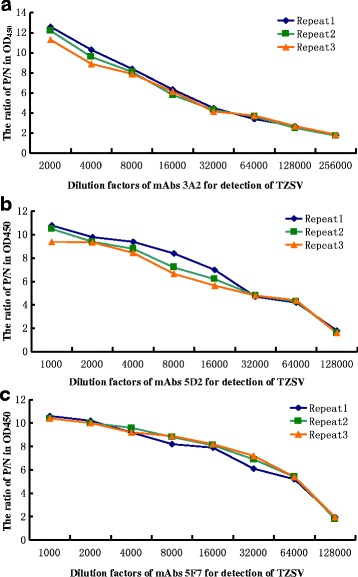
Titer of three mAbs 3A2, 5D2, 5F7 in the detection of *Tomato zonate spot virus* using the indirect ELISA. The experimental samples were used as positive (P) control, which extracted from three TZSV-infected *N. benthamian* leaves, and using healthy *N. benthamian* leaf extracts as negative (N) control. The P/N value of more than 2 indicated the positive result, and those of less than 2 represented the negative result. The X axis shows the dilution factor of mAbs 3A2, 5D2, 5F7 for TZSV and the Y axis the OD450 value for P/N

**Fig. 3:**
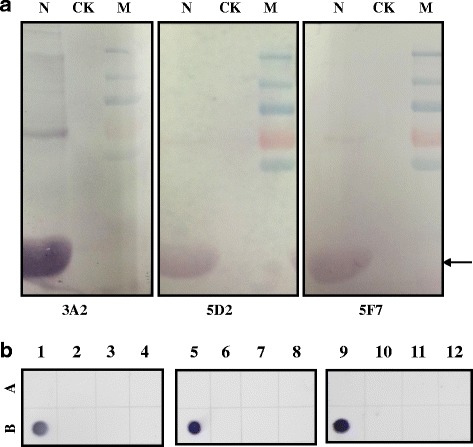
Specificity of mAbs 3A2, 5D2, 5F7 for detection of TZSV. **a** Specificity of the mAbs 3A2, 5D2, 5F7 for the detection of TZSV using western blot. The position of the TZSV-N was indicated by an arrow. M: Molecular size marker, N: the extract from TZSV-infected leaves, CK: the extract from non-infected leaves. **b** Specificity analysis of mAb 3A2 using the dot-ELISA. A1, A5, A9: negative controls; B1, B5, B9: TZSV-infected leaf extracts; A2, A6, A10: CMV-infected leaf extracts; B2, B6, B10: TMV-infected leaf extracts; A3, A7, A11: TSWV-infected leaf extracts; B3, B7, B11: INSV-infected leaf extracts; A4, A8, A12: CGMMV-infected leaf extracts

The titers of the mAbs 3A2, 5F7 and 5D2 were determined by an indirect ELISA assay using the crude extracts of TZSV-infected *N. benthamiana* leaves as coating antigen, the crude extracts from uninfected *N. benthamiana* leaves were used as the negative control. The titer of the mAbs 3A2 was up to 128,000, whereas the titers of both mAbs 5F7 and 5D2 were up to 64,000 (Fig. 2). A western blot assay was also conducted to test the specificity of the mAbs to TZSV using TZSV-infected *N. benthamiana* leaves extracts. Leaves extracts from uninfected-*N. benthamiana* leaves were used as the negative control. The result showed that the N protein of TZSV could be specifically recognized by all three mAbs (Fig. 3a). The specificity of the mAbs was further confirmed by reactions with the crude extracts from *N. benthamiana* leaves infected with five other viruses, containing *Cucumber mosaici virus* (CMV), *Tobacco mosaic virus* (TMV), *Tomato spotted wilt virus* (TSWV), *Impatiens necrotic spot virus* (INSV) and *Cucumber green mottle mosaic virus* (CGMMV) through a dot-ELISA. The results showed all the mAbs reacted specifically with the crude extracts of the TZSV-infected leaves, but did not react with the crude extracts of other five viruses-infected and uninfected leaves, indicating that all three mAbs were highly specific and sensitive for TZSV (Fig. 3b).

### Preparation of colloidal gold-labeled monoclonal antibodies

**Fig. 4:**
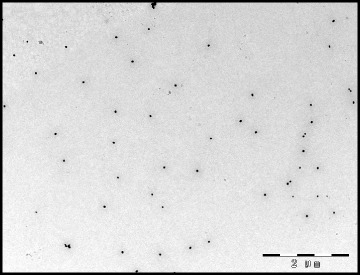
Transmission electromicroscopy (TEM) image of the colloidal gold particles. Bar = 2 μm

Colloidal gold was prepared by using trisodium citrate to reduce the chloroauric acid (at a ratio of 1:1.1 of chloroauric acid and trisodium citrate) and made the gold particles polymerizing. The prepared colloidal gold particles were observed by transmission electron microscope (TEM) (Fig. 4). The colloidal gold contained uniformly sized spheres with a diameter of approximately 30–40 nm.

**Fig. 5:**
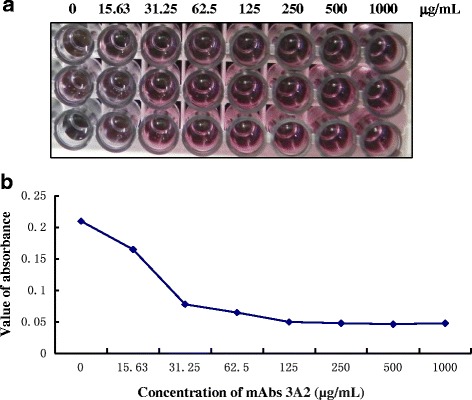
Determining the optimal ratio of colloidal gold and mAb 3A2 for conjugation. **a** A fixed amount of colloidal gold (200 μL) was mixed with an increasing amount, from 0 to 1 mg/mL (30 μL), of mAb 3A2. As the concentration of the antibodies increased, the color of the conjugated antibody-colloidal gold became pink. **b** The curves of OD528 values represented the absorbance from conjugated colloidal gold

The anti-TZSV mAb 3A2 was used to conjugate with the colloidal gold and the proportion of the colloidal gold was determined by the salt precipitation assay. The colloidal gold (125 μL) was mixed with an increasing amount of mAb 3A2. As the proportion of the antibodies increased from 0 to 1000 μg/mL, the color of the fixed solution became deep purple. When the proportion of the antibodies increased to more than 125 μg/mL (30 μL), the intensity of the purple color did not increase any further (Fig. 5a). The result of UV absorption (OD_528nm_) method showed that the absorption curve dropped when the proportion of the antibodies increased from 0 to 125 μg/mL, and the absorption curve was nearly smooth when the antibodies increased further from 125 to 250 μg/mL (Fig. 5b). These data indicated that 30 μL of 125 μg/mL antibodies was the optimized concentration for coupling with 125 μL of colloidal gold. To achieve the best result, the amount of the mAb 3A2 was increased by 10% with a final concentration of 150 μg/mL for the conjugation.

### Assembly of GICA strips for detection of TZSV

**Fig. 6:**
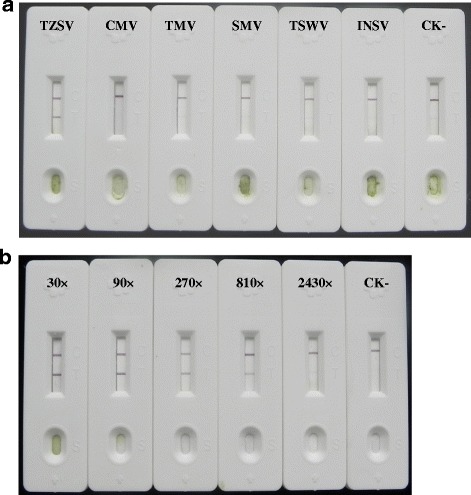
Specificity and sensitivity analysis of GICA strips for the detection of TZSV. **a** Specificity of GICA strips for the detection of TZSV. GICA strips were used to test different leaf extracts from the *Nicotiana benthamiana* plants infected with TZSV, CMV, TMV, SMV, TSWV and INSV, respectively. Leaf extract from the healthy plant was used as the negative control. **b** Sensitivity of GICA strips for the detection of TZSV. A series of diluted leaf extracts from TZSV-infected tissue were tested by GICA strips. Leaf extract from healthy plant was used as the negative control

To determine the optimal type of nitrocellulose membrane used for assembling the GICA strips, different brands, including Millipore 120, Millipore 135, Millipore 180 and Whatman, were tested for their sensitivity and performance. Millipore 180 nitrocellulose membrane was finally chosen as the best one for the detection of TZSV (data not shown). Then the strips were assembled by sequentially adding a sample absorbing pad, an application pad with conjugated mAb 3A2-colloidal gold, a Millipore 180 nitrocellulose membrane and a water absorbing pad. In order to capture the TZSV-combined monoclonal antibody 3A2-colloidal gold complexes, another mAb 5D2 was purified and coated at the test line on the nitrocellulose membrane. The test line would turn purple if the sample contained TZSV. In order to prove that gold particles coated with antibodies flowed along the strip, protein A was coated at the control line on the nitrocellulose membrane to capture all antibody-conjugated colloidal gold. TZSV-infected and -uninfected plant leaf extracts were added to the sample pad separately. The color of test line became obviously purple in the strip treated with TZSV-infected leaf sample within 5–10 min, whereas the color of the test line remained unchanged in the strip treated with TZSV-uninfected leaf sample (Fig. 6). The control lines of both strips turned purple, which indicated that the strips were valid.

### Specificity and sensitivity of the TZSV GICA strips

To determine the specificity of the TZSV GICA strips, they were tested with the *N. benthamiana* leaf extracts of the plants infected with CMV, TMV, SMV, TSWV, INSV and TZSV, respectively. Positive results were only observed in the strips tested with TZSV-infected sample but not in the stripes tested with any other viruses-infected samples (Fig. 6a). This result suggested that the strips could be used to detect TZSV specifically.

To test the sensitivity of the strips, the TZSV-infected *N. benthamiana* leaf extract was serially diluted from 1:30 to 1:2430, and 200 μL of each dilution was added to the sample pad of the strip, the uninfected leaf extract was used as the negative control. The test line of the strips turned purple strongly when the sample was diluted from 1:30 to 1:90, and the color on the test line decreased when it was further diluted from 1:270 to 1:810, whereas the color on the test line was no longer detected when it was diluted 1:2430. These results indicated that the TZSV GICA strips could still give a positive signal with an 810-fold dilution of the TZSV-infected leaf sample of *N. benthamiana* but fold less than 1:90 was the best dilution (Fig. 6b).

## Discussion

In this study, a GICA was developed based on preparation of mAbs for the rapid detection of TZSV. The analysis could be completed within 5–10 min. The detection of GICA strip specifically reacted with TZSV and no cross reaction was detected for other viruses. This was the first report of the development of a GICA for the detection of TZSV.

Prokaryotic expression was used to obtain recombinant proteins for immunogen. Compared with virus particle purification, the strategy of prokaryotic expression provided a fast approach to generate a high yield of TZSV antigens. Five mice immunized with recombinant TZSV-N all generated high-antiserum titer antibodies, the highest titer reached 40,000 dilution, suggesting that the prokaryotic expression of TZSV-N produced a high-titer antiserum. After cell fusion and screening of hybridoma cell lines, three independent cell lines of mAbs (3A2, 5D2, 5F7) against TZSV were obtained. All the three kinds of mAbs had high titer (1: 64, 000 to 1: 128, 000) in the detection of TZSV. All three mAbs only reacted with TZSV and did not react with other *Tospoviruses* or other non-related plant viruses. These mAbs would also be very useful in the detection of TZSV by dot-blot ELISA or other ELISA based approaches.

The method of sodium citrate reduction gold chloride acid was adopted to prepare colloidal gold. The more the content of sodium citrate, the smaller the size of the colloidal gold particle. By adjusting the amount of sodium citrates that were added, the size of the colloidal particles with 40 nm was obtained. The generated colloidal gold particles were uniform as checked by electromocroscopy and uniformity of colloidal gold particle was the key factor for the nature of conjugated antibody for GICA assay. To achieve the efficient conjugation of mAbs to colloidal gold particle, the ratio of antibody and colloidal gold particle was optimized. In order to reduce the coagulation of colloidal gold and the separation of colloidal gold protein complex, the right amount of stabilizer could be added into the solution, such as BSA, sodium borate, PEG, etc. In the test of a flatoxin by colloidal gold province immune chromatography, Deng et al [41] used 0.1% of PEG and 1% of BSA as stabilizer. In the detection of chlorpyrifos-methyl in water samples by an immuno chromatographic assay, Hua et al [42] used 0.1% of sodium borate as stabilizer. This study used sodium borate as stabilizer and got satisfied results.

The assembled GICA strip could specifically recognize the TZSV, without cross reaction with CMV, TMV, SMV, TSWV and INSV. The sensitivity of the GICA strip had the detection limit of 1:810 diluted from sap of the TZSV-infected leaf tissue but the fold less than 1:90 was the optimized dilution. Compared with ELISA, western-blot, RT-PCR and electron microscopy technology, GICA for the detection of TZSV had several advantages: 1) the analysis process could be completed in shorter time (5–10 min); 2) the operation procedure was very simple and did not require professional training; 3) the testing results were visible to the naked eye and the assay did not rely on any special equipment; 4) the detection for TZSV was specific and relatively sensitive. All these advantages and the simplicity of the operation made it especially suitable for rapid diagnosis and immediate surveillance of TZSV-induced diseases in the field.

## Conclusions

TZSV was an Asia type tospoviruses and caused significant losses in many vegetables and orgamental crops in Southwest China. In this study, we obtained three mAbs and developed a simple, convenient and rapid GICA specific for TZSV detection. The obtained mAbs and GICA had a great potential in application of the rapid detection and surveillance of TZSV.

## Abbreviations

ELISA: Enzyme-linked immunosorbent assay
GICA: Colloid gold immunochromatographic
MAbs: Monoclonal antibodies
NSm protein: Non-structural protein
RdRp: RNA-dependent RNA polymerase
RT-PCR: Reverse transcription polymerase chain reaction amplification
SDS-PAGE: Sodium dodecyl sulfate-polyacrylamide gel
ss RNA: Single-stranded RNA
TZSV: *Tomato zonate spot virus*
